# Development of a clinical decision support system for diabetes care: A pilot study

**DOI:** 10.1371/journal.pone.0173021

**Published:** 2017-02-24

**Authors:** Livvi Li Wei Sim, Kenneth Hon Kim Ban, Tin Wee Tan, Sunil Kumar Sethi, Tze Ping Loh

**Affiliations:** 1 Department of Biochemistry, National University of Singapore, Singapore; 2 Department of Laboratory Medicine, National University Hospital, Singapore; University of Michigan, UNITED STATES

## Abstract

Management of complex chronic diseases such as diabetes requires the assimilation and interpretation of multiple laboratory test results. Traditional electronic health records tend to display laboratory results in a piecemeal and segregated fashion. This makes the assembly and interpretation of results related to diabetes care challenging. We developed a diabetes-specific clinical decision support system (Diabetes Dashboard) interface for displaying glycemic, lipid and renal function results, in an integrated form with decision support capabilities, based on local clinical practice guidelines. The clinical decision support system included a dashboard feature that graphically summarized all relevant laboratory results and displayed them in a color-coded system that allowed quick interpretation of the metabolic control of the patients. An alert module informs the user of tests that are due for repeat testing. An interactive graph module was also developed for better visual appreciation of the trends of the laboratory results of the patient. In a pilot study involving case scenarios administered via an electronic questionnaire, the Diabetes Dashboard, compared to the existing laboratory reporting interface, significantly improved the identification of abnormal laboratory results, of the long-term trend of the laboratory tests and of tests due for repeat testing. However, the Diabetes Dashboard did not significantly improve the identification of patients requiring treatment adjustment or the amount of time spent on each case scenario. In conclusion, we have developed and shown that the use of the Diabetes Dashboard, which incorporates several decision support features, can improve the management of diabetes. It is anticipated that this dashboard will be most helpful when deployed in an outpatient setting, where physicians can quickly make clinical decisions based on summarized information and be alerted to pertinent areas of care that require additional attention.

## Introduction

Diabetes is a chronic metabolic disorder that is characterized by persistent elevation of blood glucose. Globally, it is a healthcare priority that affects 366 million people in 2011, and this is projected to increase to 552 million by 2030 [1]. The management of diabetes is highly complex as it is associated with complications that affect multiple organs, including retinopathy, nephropathy, foot ulcers and autonomic nerve dysfunction. Diabetes is also associated with hyperlipidemia and an increased risk of cardiovascular disease, peripheral vascular diseases and stroke, which account for most of the mortality in patients with diabetes [2].

Management of diabetes is guided by evidence-based clinical recommendations [3]. Patients with diabetes need to be treated to meet certain targets, which are based on laboratory assessment, to minimize the risk of development of long-term complications. Hence, they are monitored by regular measurement of laboratory markers related to glycemic control, lipid control and renal function [3]. The glycemic control can be assessed by fasting plasma glucose and glycated hemoglobin A_1c_ (HbA_1c_), whereas lipid control can be monitored by serum total cholesterol, high-density lipoprotein (HDL), low-density lipoprotein (LDL) and triglycerides. The renal function is monitored by estimated glomerular filtration rate (eGFR) and urinary albumin:creatinine ratio (UACR). Each of these laboratory tests has its own recommended target that guides clinicians on treatment adjustment, and should be repeated at certain time intervals [3]. This makes monitoring of these laboratory markers a highly complex cognitive task.

There is evidence to show that monitoring of the diabetes-related markers is not performed optimally in routine clinical settings. For example, a study in England examined approximately half a million HbA_1c_ measurements and found that 21% of the assessments were done too frequently, and 30% were done too infrequently [4]. In Singapore, Loh and colleagues [5] found that 51% of HbA_1c_ measurements were repeated before the recommended three-month testing interval at a large tertiary-care teaching hospital. The under- and over-utilization of tests is a major problem as inappropriate monitoring of HbA_1c_ outside of guidelines is associated with a significant impairment in the control of diabetes [4].

A possible contributing factor to this sub-optimal testing could be the conventional way of presenting laboratory results, which are often piecemeal and segregated. A clinical data interface that does not effectively consolidate data or provide recommendation and reminders to clinicians might increase the cognitive burden during a clinical consultation, and contribute to errors in the disease monitoring process.

CDSSs can be designed to alleviate this problem by integrating up-to-date patient data and specific clinical information (e.g. evidence-based guidelines), to generate patient-specific reports or dashboards where the information is summarized and analyzed. [6]. In doing so, the user is provided with easy to read results and disease-specific knowledge that includes clear, actionable recommendations [7, 8]. With the automation of such mentally taxing tasks, physicians may be able to reduce the time taken to interpret the laboratory result and focus on other areas of care [9]. Furthermore, physicians’ clinical skills may also be improved by learning from the corrective messages supplied by the system [10].

The current laboratory result reporting system in use in the National University Hospital, Singapore, displays results related to diabetes care in separate sections, and without active decision support capabilities. The scattered nature of the laboratory results makes the assembly and interpretation of results related to diabetes care challenging. We hypothesized that an integrated CDSS interface for presenting laboratory data can enhance their interpretation.

Hence, the objectives of this study are:

1. To design and develop a diabetes-specific CDSS interface for displaying glycemic, lipid and renal panel results, in an integrated form with decision support capabilities,
2. To determine if the use of this CDSS interface, compared to the existing laboratory reporting interface, results in improved interpretation of laboratory results.

## Materials and methods

### Current laboratory reporting system in use

**Fig 1:**
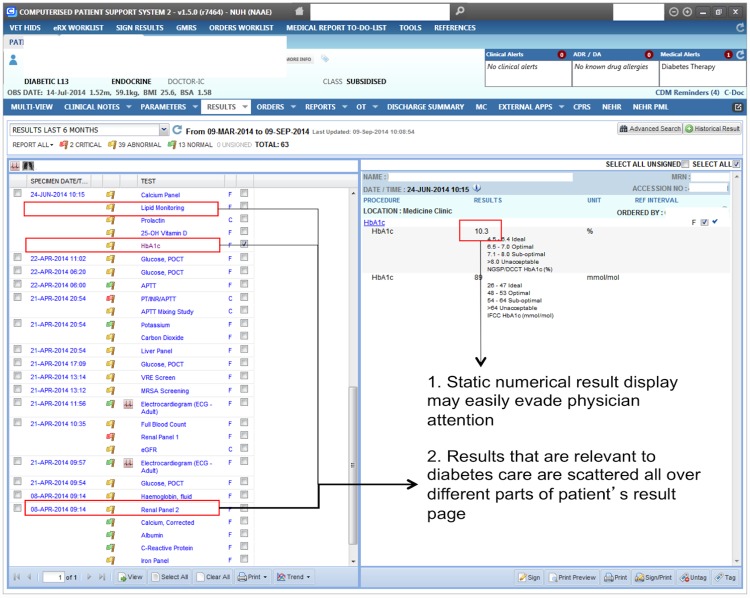
A screenshot of Computerized Patient Support System 2 showing the HbA_1c_ result of a patient as an example. The left panel displays a list of all the historical laboratory and radiological tests ordered for a patient. The right panel displays the results of the individual test order on the left panel.

The Computerized Patient Support System 2 (CPSS2) is a personal computer-based electronic health record system that is currently in use in the National University Hospital, Singapore. It is locally developed by the Integrated Health Information Systems agency. Under this system, laboratory results related to diabetes care, i.e. HbA_1c_, the lipid panel, and the renal panel, are displayed in a list along with results from other tests, and each result or panel must be viewed individually (Fig 1). Hence, multiple clicks are required to access historical or different laboratory results.

### Design and development of the clinical decision support system interface

**Fig 2:**
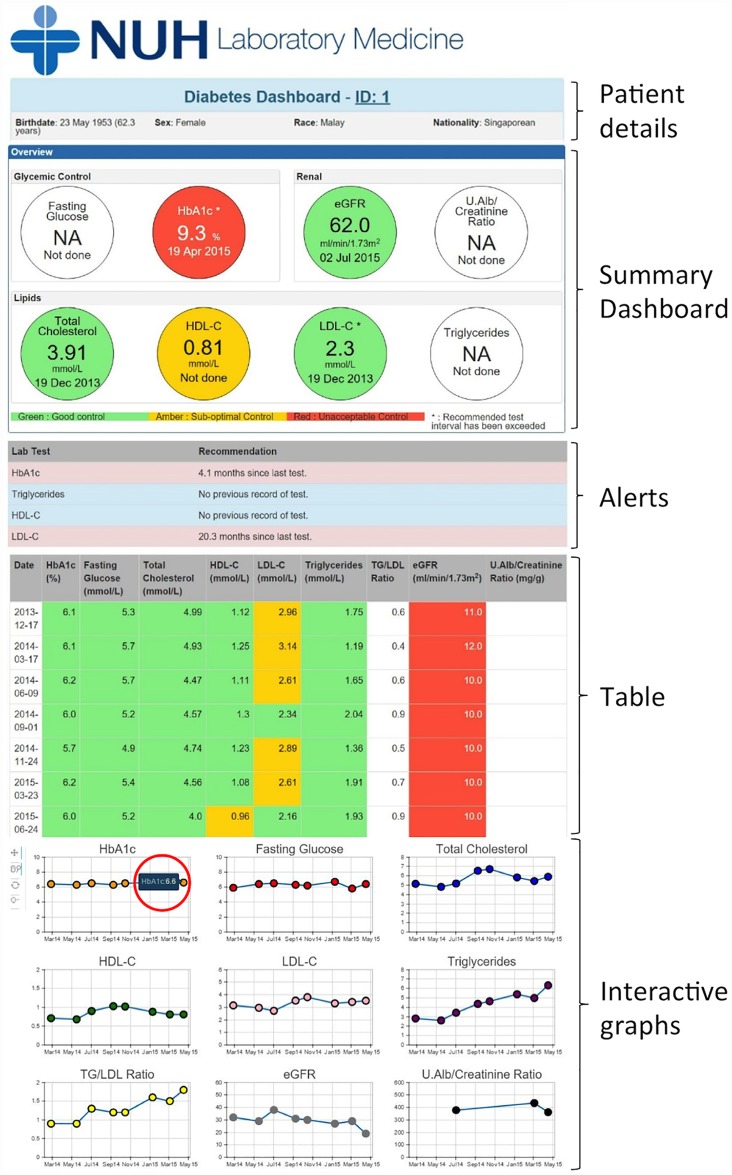
A screenshot of the summary dashboard, alerts, table and interactive graphs in the Diabetes Dashboard. An example of the hover tool is shown, displaying the test result of the individual point in the HbA_1c_ graph when the mouse hovers over it (circled in red).

The CDSS, which we named the “Diabetes Dashboard”, is a web application that can be accessed through an Internet browser (Fig 2). It was designed in collaboration with practicing physicians with the aim of displaying laboratory results related to diabetes care in an integrated manner, together with appropriate alerts to flag abnormal values and sub-optimal testing intervals, which would support clinicians in their management of diabetes and its associated risk factors. Under this data display algorithm, all laboratory results related to diabetes are simultaneously displayed at a single click.

MySQL (https://www.mysql.com/), HTML, CSS and Bootstrap (http://getbootstrap.com/) were used to develop the web-based interface of the DD. The Python programming language (https://www.python.org/) was used to develop the various CDSS tools, such as the color-coding module, the alert module and the interactive graph module. For flexibility, all target values were specified in a human-readable configuration file, which is used by the CDSS tools to produce the appropriate colors or alerts. The treatment targets and testing intervals for the laboratory tests were extracted from the Clinical Practice Guidelines published by the Ministry of Health (Singapore) (https://www.moh.gov.sg/cpg).

#### Color-coding module

A color-coded module, based on the recommended treatment targets for glycemic, lipid and renal panels by the Ministry of Health, Singapore, was designed to indicate different levels of control of the various laboratory markers related to diabetes care (Table 1). If the test result indicates ideal or optimal control, it is colored green. On the other hand, if the test result indicates sub-optimal control or poor control, the test result is colored amber or red, respectively.

**Table 1:** Treatment targets for each laboratory marker related to diabetes care, and their assigned color codes.

Test	Good control Green	Intermediate control Amber	Poor control Red
Fasting Glucose (mmol/L)	≤ 8.0	8.1–10	> 10
HbA1c (%)	≤ 7.0	7.1–8.0	> 8.0
Total Cholesterol (mmol/L)	< 5.2	5.2–6.1	> 6.1
HDL Cholesterol (mmol/L)	≥ 1.0	-	< 1.0
LDL Cholesterol (mmol/L)	< 2.6	2.6–4.1	> 4.1
Triglycerides (mmol/L)	< 2.3	2.3–4.5	> 4.5
Urine Albumin: Creatinine Ratio (mg/g)	< 30	30–300	> 300
eGFR (mL/min/1.73m^2^)	> 60	30–60	< 30

#### Alert module

Another feature of the CDSS, the alert module, is used to indicate that a particular test has exceeded the recommended testing interval. This module was programmed to calculate the number of months that has elapsed from the latest test performed, compared to the present date. These alerts were similarly designed based on the recommended testing intervals for diabetes-related disease monitoring by Ministry of Health (Table 2).

**Table 2:** Testing intervals for each laboratory marker related to diabetes care used in the alert module.

Test	Repeat 3 monthly	Repeat 6 monthly	Repeat yearly
**Fasting Glucose (mmol/L)**	-	-	Yes
**HbA1c (%)**	If HbA1c > 7.0	If HbA1c ≤ 7.0	-
**HDL Cholesterol (mmol/L)**	-	If HDL < 1.0	If HDL ≥ 1.0
**LDL Cholesterol (mmol/L)**	-	If LDL > 2.6	If LDL ≤ 2.6
**Triglycerides (mmol/L)**	-	If TG > 2.3	If TG ≤ 2.3
**Urine Albumin: Creatinine Ratio (mg/g)**			Yes
**eGFR (ml/min/1.73m2)**			Yes

#### Interactive time-series graphs

Interactive graphs were generated for each of the laboratory tests, with the use of Bokeh (bokeh.pydata.org/), an interactive visualization library in Python. Using all historical test results, these graphs were plotted using test results with test dates as a function.

### Features displayed in the Diabetes Dashboard

#### Patient information

This displays the information of the patient, such as the age, date of birth, race and nationality of a patient. For the purpose of this study, patient identifiers such as name and personal identification number were not included as part of the patient information. These can be easily included when necessary.

#### Summary dashboard

The summary dashboard is divided into the glycemic panel (HbA_1c_ and fasting plasma glucose), renal panel (eGFR and UACR) and lipid panel (total cholesterol, HDL, LDL and triglycerides) (Fig 2). Each circle displays the latest result for each of the laboratory markers related to diabetes care, and is color-coded according to the level of control of the markers. The interpretation of these color codes is shown in the legend located at the bottom of the summary dashboard. Another decision support feature is the asterisk, which is displayed next to the test name. It appears when the recommended testing interval has been exceeded for that particular test.

#### Alerts

Using the alert module, alerts are generated when a particular test has exceeded the recommended testing interval. The number of months since the last test is displayed next to the test name (Fig 2). These alerts serve as a reminder to the user that these tests should be repeated soon. Alerts are also generated when there is no previous record of a particular test.

#### Table

The table displays all past laboratory results in chronological order (Fig 2). Similar to the summary dashboard, the results are color-coded according to the level of control of the laboratory markers related to diabetes care, using the color-coding module.

#### Interactive time-series graphs

Using the laboratory results of a patient, interactive graphs are generated for each of the tests (Fig 2). The test results are plotted against test dates, allowing the user to view any long term trends that may be present. Graph tools located on the left side of the graph tool allow the user to pan, zoom and reset the graphs when required. Additionally, the panning of all the graphs along the x axis is synchronized, in order to allow a direct comparison of the results. Users can also view the test result for each individual point in the graphs by hovering over it.

### Evaluation of the Diabetes Dashboard

An online self-administered survey was designed to compare the ease of detecting abnormal values, suboptimal testing periods, and long-term trends, as well as of identifying the need for treatment adjustment between the Diabetes Dashboard and a mock version of the CPSS2 interface. This was conducted using FluidSurveys (https://fluidsurveys.com/), an online survey tool that allows for randomization and timed surveys.

#### Survey participants

An open invitation to participate in this survey was extended via email to the final year medical students (N = 300), who were undergoing their final year examinations from the Yong Loo Lin School of Medicine, National University of Singapore. Thirty-eight of these students volunteered to participate in the survey and provided their consent electronically by clicking a consent checkbox before the start of the online survey. This survey was conducted anonymously. The final year medical students were chosen as study subjects as they were expected to have the clinical knowledge of a junior doctor, who is expected to manage diabetes, and were relatively naive to any electronic health record systems.

#### Survey design

**Fig 3:**
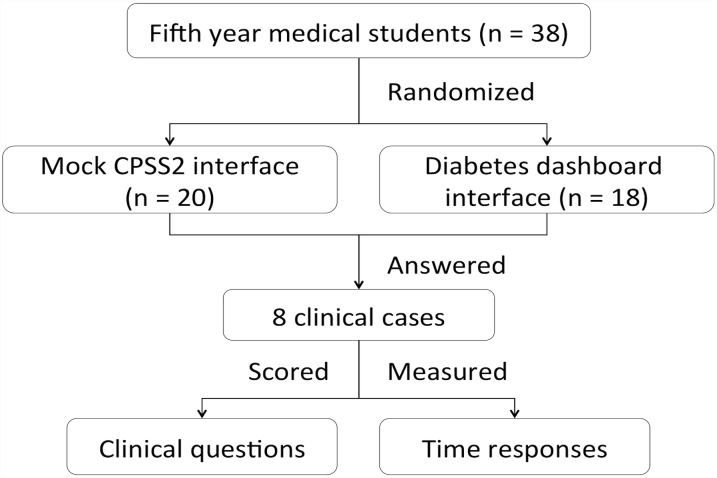
Flowchart summarizing the overall design of the survey.

A summary of the survey design is shown in Fig 3. Survey participants were randomly assigned to either the Diabetes Dashboard or the mock CPSS2 interface. Eight clinical scenarios were displayed in their assigned interface, and the participants had to answer a series of clinical questions regarding the detection of abnormal values, suboptimal testing periods, long term trends, and identifying the need for treatment adjustment for each of the scenarios. Each student saw the same 8 cases that each other student saw. The clinical scenarios were jointly designed by two consultant clinicians. The complete set of survey questions are provided as S1 Text. The time the participants spent on each case was also recorded.

#### Mock CPSS2 interface

**Fig 4:**
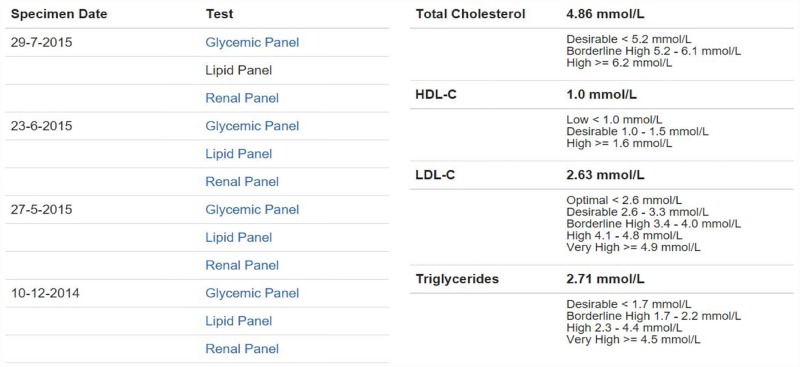
An example of the lipid panel displayed in the mock CPSS2 interface.

The mock CPSS2 interface was designed to closely mimic the layout of the CPSS2 system. The HTML styles for both the Diabetes Dashboard and the mock CPSS2 were standardized to eliminate this as a confounding factor in user interface perception (Fig 4).

### Analysis of the survey results

A scoring system was used to grade the answers of each participant. For each correct answer, a point was awarded. The cumulative points achieved by a participant were then used to calculate the total percentage score for each question. The nonparametric Mann-Whitney U test was used to compare the differences in scores and time taken between the two groups of participants, where a p-value of <0.05 was considered as significant. All the statistical analysis was performed using Graphpad prism 6 (http://www.graphpad.com/scientific-software/prism/).

## Results

### Perceived familiarity with local clinical practice guidelines

**Fig 5:**
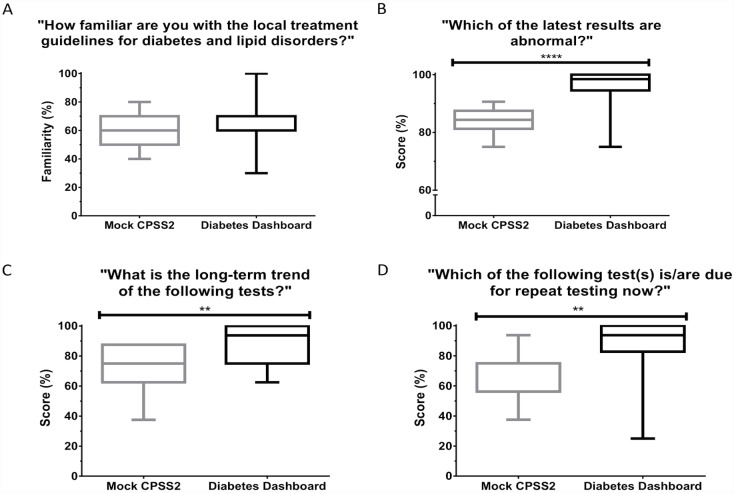
Box plots showing the participants’ (A) perceived familiarity with diabetes-related guidelines, (B) ability to identify the abnormal test values in the most recent glycemic panel results, (C) ability to identify the long-term trends of results of the markers in the glycemic panel, (D) ability to identify if the HbA_1c_ or LDL tests needed to be retested, using either interface. The exact question asked in the survey is shown in the title of the graphs. ** denotes *p*<0.05, **** denotes *p*<0.0001.

To ensure that the two randomized groups of participants had comparable confidence in their baseline knowledge, we surveyed their perceived familiarity with diabetes-related clinical practice guidelines. There was no significant difference between the two groups in terms of perceived familiarity and background knowledge that could have confounded the survey results. Participants using the Diabetes Dashboard and mock CPSS2 interfaces expressed a mean percentage familiarity of 62% and 63% (*p* = 0.64), respectively (Fig 5).

### Identification of abnormal results in the glycemic panel

When the participants were asked to identify which of the test results shown had sub-optimal or poor control, the participants using the Diabetes Dashboard scored on average 11% higher than the mock CPSS group (96% vs. 85%, *p*<0.0001) (Fig 5).

### Identification of long-term trends of markers in glycemic panel

The participants were asked to identify the long-term trends of results of the glycemic panel, using their assigned interface. Fig 5 shows the scores of the participants using the Diabetes Dashboard and the mock CPSS2 interfaces. The participants using the Diabetes Dashboard scored 17% higher, compared to the participants using the mock CPSS2 interface (89% vs 72%, *p* = 0.0013).

### Identification of need for repeat testing of HbA_1c_ and LDL

For this question, participants had to identify whether the time elapsed between the last HbA_1c_ or LDL tests and the present date has exceeded the recommended testing intervals, and if so, to recognize that the tests need to be repeated. Fig 5 shows that the participants using the Diabetes Dashboard scored significantly better compared to the participants using the MC interface (85% vs 65%, p = 0.0023).

### Identification of need for treatment adjustment for diabetes and cholesterol

**Fig 6:**
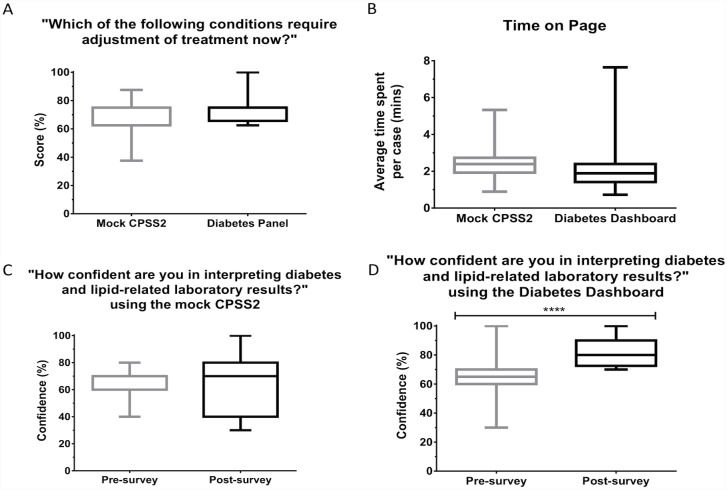
Box plots showing the participants’ (A) ability to determine if the adjustment of treatment was required, (B) average time (in minutes) spent on each case, (C) perceived confidence before and after completing the survey using the mock CPSS2 interface, (D) perceived confidence before and after completing the survey using the mock CPSS2 interface, using either interface. The exact question asked in the survey is shown in the title of the graphs. ** denotes *p*<0.05, **** denotes *p*<0.0001.

Participants were asked to interpret the tests' results and determine if adjustment of treatment was needed for diabetes or cholesterol. The score of the participants are shown in Fig 6. Although participants using the Diabetes Dashboard were found to have an 8.75% higher mean scores than participants using the mock CPSS2 interfaces, this difference was not significant (*p* = 0.0696).

### Comparison of the time spent on each case

The time taken for participants to complete each case was tracked by the survey tool. While the participants using the Diabetes Dashboard (2.12 minutes) spent on average 0.51 minutes less time per case compared to participants using the mock CPSS2 interfaces 316 (2.63 minutes), this difference was not found to be significant (p = 0.08, Fig 6).

### Perceived confidence in interpreting diabetes-related laboratory results

Participants were asked to rate their confidence in interpreting diabetes-related test results, before and after the survey. While we did not see a significant difference in confidence for the participants using the mock CPSS2 interfaces, there was a significant increase in confidence for the participants using the Diabetes Dashboard after the survey compared to before the survey (Fig 6).

## Discussion

CDSSs can feature a wide assortment of functions that aid in decision-making. Some examples are patient data reports, reminders and alerts, clinical guidelines, diagnostic support, and tools for clinical workflow [11]. Decision support tools can be classified into three tiers [10]. Tools in the first tier are used to manage clinical information. These include the automated retrieval of patient information and the filtration of data to produce patient data reports or dashboards, as well as simple calculations of examination results. In the second tier, the tools aim to grab the attention of the user, such as those that flag abnormal results, and give brief alerts when there is an uncompleted task. Lastly, the tools in the third tier use patient data to generate patient-specific recommendations and clinical advice. These advice messages are based on specific algorithms, clinical pathways and guidelines, or cost-benefit analysis [12].

The CDSS developed in this study represents a tier 2 tool since it included features that extract the laboratory data, display them in a summarized dashboard format, and provide alerts for abnormal result and retesting. This CDSS does not provide patient-specific recommendations or diagnosis, as required to be considered a tier 3 CDSS [10].

The rapid rise in diabetes burden coupled with limited healthcare resources in an austere environment has made CDSS an attractive tool to improve delivery of care in a scalable manner [13–17]. CDSS designs can differ significantly in content and scope [18]. They have been used to automate test and treatment recommendations [19,20], assist in risk stratification for diabetic foot screening [21], promote health communication with patients [22], predict blood glucose [23], interpret self-monitoring of blood glucose data [24,25], monitor guideline adherence [26], correct/ prevent medication error [27], and detect potential adverse drug interactions [28].

Studies examining the effectiveness of CDSSs in managing chronic diseases have produced mixed results. In general, systematic reviews have found that they significantly improve process outcomes (e.g. increasing laboratory testing rate, foot screening rate) [8,15–17,29]. However, they have weak to moderate effects (which are often statistically not significantly different from the control group) on commonly monitored clinical outcomes such as improvement in biochemical parameters, (reduced) use of insulin sliding scale, (increased) use of basal-bolus insulin regime, quality of life and hospitalization [29–33]. At least one systemic review has found the use of CDSSs does not affect patient mortality rates [34].

While the heterogeneity of published studies on CDSSs makes pooled analysis of the clinical outcomes challenging, several limitations have been noted to reduce the effectiveness of CDSSs. They include the inconsistent use of CDSSs, poor adherence to alerts, lack of integration of CDSSs into clinical workflow, and the inability of CDSSs to innately foster collaborations with patients to improve compliance [29,32]. Often, the social, organizational and contextual characteristics are overlooked during the design and implementation phases of CDSS [32]. In primary care setting, CDSSs are most effective when combined with feedback on performance and case management [35].

The cost-effectiveness of a CDSS needs to be carefully weighed against its potential benefits, particularly when its impact on clinical outcomes is not universally positive and large [36,37]. Indeed, some studies have found that the cost of implementation of a CDSS can be larger than any potential cost savings from improvement in short-term risk factors or higher detection rate of complications [37,38]. Others have demonstrated potential cost savings [36]. Low- to medium-income countries should be mindful about cost-effectiveness as evidence of the benefits for implementing CDSSs is lacking from these regions [29].

In this randomized, self-administered survey on the final year medical students, we found that both the Diabetes Dashboard and mock CPSS2 groups reported similar levels of familiarity and knowledge of diabetes-related guidelines at baseline. This indicates that the groups were well randomized in terms of their background knowledge, and minimized any potential confounding effects from these factors in the performance of the survey.

In this study, the Diabetes Dashboard interface had better (higher) the scores for (i) detecting abnormal results, (ii) identifying long-term trends, and (iii) identifying the need for retesting for the various markers in the diabetes panel. These results support the hypothesis that a CDSS could increase the ease of interpretation, and is consistent with previous studies demonstrating that CDSS can improve the monitoring of therapy [39], and process of care [40].

Significantly, our results show that the Diabetes Dashboard interface resulted in a significant increase in participants’ awareness of when they should repeat tests, compared to the participants using the mock CPSS2 interface. This could be attributed to the inclusion of an alert module in the Diabetes Dashboard interface that highlights testing intervals, thus reducing the attention load for identifying the need for repeat testing. Our findings are consistent with previous studies reporting that testing behavior for disease monitoring and therapy monitoring improved by 63% and 35% respectively, with the use of CDSSs that incorporate alerts [41].

Interestingly, in this study, the Diabetes Dashboard interface did not appear to have a significant impact on the performance of the participants in determining whether adjustment of treatment was required. It is possible that this could be due to the lack of familiarity of the participants with the treatment targets contained in the local clinical practice guidelines. This postulation is supported by the relatively low self-reported confidence and familiarity with the local clinical practice guidelines, and the low scores achieved for answering questions related to treatment targets at the start of the survey.

This is not entirely unexpected given that the participants are final year medical students who are not yet actively managing diabetic patients. Thus, although the Diabetes Dashboard interface can help in the interpretation of results, a lack of treatment-specific knowledge recommended by the guidelines will still limit the effectiveness of the management of diabetes. This finding indicates that decision support system must be integrated with sound medical management knowledge and principles for optimal practice.

Interestingly, we found a significant increase in the confidence in assessing laboratory results related to diabetes care for participants using the Diabetes Dashboard after the survey. There was no difference in confidence for participants using the mock CPSS2 interface. This suggests that the Diabetes Dashboard interface was perceived to be a reliable evidence-based decision support system for assessing diabetes-related results. However, this increased confidence may become a potential problem when users develop an over-reliance on decision support systems, resulting in expectations of infallibility of the system, or users not being able to work efficiently without these systems [42].

There are several limitations in this study. Firstly, the response rate for the survey was rather low (13%) but this limitation was mitigated by the randomized study design. Nevertheless, the results of the surveys are underpowered and may represent a unique cohort of students. They should be interpreted with caution. Moreover, the advantages of the dashboard design may be diminished in experienced physicians, who are better at interpreting laboratory results. We recognize that our instructions to participants (see Supporting Information) might have suggested to the students that the new dashboard is a better interface compared to the CPSS2 interface. It would have been better not to convey any judgments regarding the task to which the student is assigned.

In conclusion, we have developed and shown that the use of the Diabetes Dashboard, which incorporates several decision support features, can improve the management of diabetes. It is anticipated that this dashboard will be most helpful when deployed in an outpatient setting, where physicians can quickly make clinical decisions based on summarized information and be alerted to pertinent areas of care that require additional attention.

## Supporting information

S1 Text Complete set of survey questions. (DOC)
