# Supplementary material for: Development of a clinical decision support system for diabetes care: A pilot study
Source: PLoS One. 2017 Feb 24;12(2):e0173021. doi: 10.1371/journal.pone.0173021 (PMC5325565; doi:10.1371/journal.pone.0173021)
Supplement: S1 Text — (DOC) [file pone.0173021.s001.doc]

Online survey form

Diabetes Interface Survey

Estimated time-taken: less than 15 minutes

Survey Objective:

We have designed a clinical decision support interface to aid the interpretation of diabetes- and lipid-related clinical results. We would like to find out how using this new interface in contrast to the CPSS2 interface will affect the diagnostic behaviour of physicians.

Instructions

First, you will be asked about your current knowledge of diabetes- and lipid-related treatment targets and testing intervals. Please do this from memory and do not refer to any external source. Subsequently, you will be randomly assigned to either the mock CPSS2 interface or the new dashboard interface. Next, you will be given 8 simple clinical cases. For each of them, please click on the link that will take you to the interface webpage. Please answer the questions for each case. Please note that the time spent on each page/case will be recorded for analysis purposes. There will be a link to a cheat sheet with details on the relevant testing ranges and intervals that are needed to interpret the test results. Please note that you may not return to the previous cases once you click "Next". The results of this survey are analysed anonymously and do not affect your academic grading.

1. How confident are you in interpreting diabetes and lipid-related laboratory results?

|  | 0 | 1 | 2 | 3 | 4 | 5 | 6 | 7 | 8 | 9 | 10 |  |
| --- | --- | --- | --- | --- | --- | --- | --- | --- | --- | --- | --- | --- |
| Not Confident |  |  |  |  |  |  |  |  |  |  |  | Very Confident |

2. How familiar are you with the local treatment guidelines for diabetes and lipid disorders?

|  | 0 | 1 | 2 | 3 | 4 | 5 | 6 | 7 | 8 | 9 | 10 |  |
| --- | --- | --- | --- | --- | --- | --- | --- | --- | --- | --- | --- | --- |
| Not Familiar |  |  |  |  |  |  |  |  |  |  |  | Very Familiar |

3.1 Introduction to the mock CPSS2 interface

You have been assigned to the mock CPSS2 interface.

Here are the features of this interface:

Default Page

List of dates with clickable links to the Glycemic, Lipid and Renal Panels

Glycemic Panel

Displays the test results for HbA1c and Fasting Glucose (if they are available)

Lipid Panel

Displays the test results for Total Cholesterol, HDL-C, LDL-C, Triglycerides (if they are available)

Renal Panel

Displays the test results for eGFR and Urine Albumine/Creatinine (if they are available)

3.2 Introduction to the Diabetes Dashboard

You have been assigned to the dashboard interface.

Here are the features of this interface:

Dashboard

Displays the latest test results

Color coded according to test results (legend at the bottom)

Divided into Glycemic, Renal and Lipid Panel

Alerts

Alerts for tests that have exceeded the recommended testing interval, and should be considered for retesting

Color coded:

Red: retesting due

Blue: test not done yet, consider testing

Table

Previous test results

Color coded according to test results (legend in dashboard)

Graphs

Test results plotted against test dates

Tools on the left hand side to pan, zoom and reset the graphs

3.3 Mock clinical cases

Case 1 of 8

1. Please comment on the laboratory results above.

2. Which of the following test(s) is/are due for repeat testing now?

|  | HbA1c |
| --- | --- |
|  | LDL Cholesterol |

3. If they are not due now, when would you repeat them?

| HbA1c (in months) |  |
| --- | --- |
| LDL Cholesterol (in months) |  |

Case 2 of 8

1. Please comment on the laboratory results above.

2. Which of the following test(s) is/are due for repeat testing now?

|  | HbA1c |
| --- | --- |
|  | LDL Cholesterol |

3. If they are not due now, when would you repeat them?

| HbA1c (in months) |  |
| --- | --- |
| LDL Cholesterol (in months) |  |

Case 3 of 8

1. Please comment on the laboratory results above.

2. Which of the following test(s) is/are due for repeat testing now?

|  | HbA1c |
| --- | --- |
|  | LDL Cholesterol |

3. If they are not due now, when would you repeat them?

| HbA1c (in months) |  |
| --- | --- |
| LDL Cholesterol (in months) |  |

Case 4 of 8

1. Please comment on the laboratory results above.

2. Which of the following test(s) is/are due for repeat testing now?

|  | HbA1c |
| --- | --- |
|  | LDL Cholesterol |

3. If they are not due now, when would you repeat them?

| HbA1c (in months) |  |
| --- | --- |
| LDL Cholesterol (in months) |  |

Case 5 of 8

1. Which of the latest results are abnormal?

|  | HbA1c |
| --- | --- |
|  | Fasting Glucose |
|  | Total Cholesterol |
|  | HDL Cholesterol |
|  | LDL Cholesterol |
|  | Triglycerides |
|  | eGFR |
|  | Urine Albumine/Creatinine Ratio |

2. What is the long-term trend of the following tests?

| HbA1c | |  | Increasing trend | | --- | --- | |  | Decreasing trend | |  | No significant change | |  | Increasing then decreasing trend | |  | Decreasing then increasing trend | |
| --- | --- | --- | --- | --- | --- | --- | --- | --- | --- | --- | --- |
| Total Cholesterol | |  | Increasing trend | | --- | --- | |  | Decreasing trend | |  | No significant change | |  | Increasing then decreasing trend | |  | Decreasing then increasing trend | |

3. Which of the following conditions require adjustment of treatment now?

|  | Diabetes |
| --- | --- |
|  | Cholesterol |

4. Which of the following test(s) is/are due for repeat testing now?

|  | HbA1c |
| --- | --- |
|  | LDL Cholesterol |

5. If they are not due now, when would you repeat them?

| HbA1c (in months) |  |
| --- | --- |
| LDL Cholesterol (in months) |  |

Case 6 of 8

1. Which of the latest results are abnormal?

|  | HbA1c |
| --- | --- |
|  | Fasting Glucose |
|  | Total Cholesterol |
|  | HDL Cholesterol |
|  | LDL Cholesterol |
|  | Triglycerides |
|  | eGFR |
|  | Urine Albumine/Creatinine Ratio |

2. What is the long-term trend of the following tests?

| Total Cholesterol | |  | Increasing trend | | --- | --- | |  | Decreasing trend | |  | No significant change | |  | Increasing then decreasing trend | |  | Decreasing then increasing trend | |
| --- | --- | --- | --- | --- | --- | --- | --- | --- | --- | --- | --- |
| Triglycerides | |  | Increasing trend | | --- | --- | |  | Decreasing trend | |  | No significant change | |  | Increasing then decreasing trend | |  | Decreasing then increasing trend | |

3. Which of the following conditions require adjustment of treatment now?

|  | Diabetes |
| --- | --- |
|  | Cholesterol |

4. Which of the following test(s) is/are due for repeat testing now?

|  | HbA1c |
| --- | --- |
|  | LDL Cholesterol |

5. If they are not due now, when would you repeat them?

| HbA1c (in months) |  |
| --- | --- |
| LDL Cholesterol (in months) |  |

Case 7 of 8

1. Which of the latest results are abnormal?

|  | HbA1c |
| --- | --- |
|  | Fasting Glucose |
|  | Total Cholesterol |
|  | HDL Cholesterol |
|  | LDL Cholesterol |
|  | Triglycerides |
|  | eGFR |
|  | Urine Albumine/Creatinine Ratio |

2. What is the long-term trend of the following tests?

| Triglycerides | |  | Increasing trend | | --- | --- | |  | Decreasing trend | |  | No significant change | |  | Increasing then decreasing trend | |  | Decreasing then increasing trend | |
| --- | --- | --- | --- | --- | --- | --- | --- | --- | --- | --- | --- |
| eGFR | |  | Increasing trend | | --- | --- | |  | Decreasing trend | |  | No significant change | |  | Increasing then decreasing trend | |  | Decreasing then increasing trend | |

3. Which of the following conditions require adjustment of treatment now?

|  | Diabetes |
| --- | --- |
|  | Cholesterol |

4. Which of the following test(s) is/are due for repeat testing now?

|  | HbA1c |
| --- | --- |
|  | LDL Cholesterol |

5. If they are not due now, when would you repeat them?

| HbA1c (in months) |  |
| --- | --- |
| LDL Cholesterol (in months) |  |

Case 8 of 8

1. Which of the latest results are abnormal?

|  | HbA1c |
| --- | --- |
|  | Fasting Glucose |
|  | Total Cholesterol |
|  | HDL Cholesterol |
|  | LDL Cholesterol |
|  | Triglycerides |
|  | eGFR |
|  | Urine Albumine/Creatinine Ratio |

2. What is the long-term trend of the following tests?

| HDL Cholesterol | |  | Increasing trend | | --- | --- | |  | Decreasing trend | |  | No significant change | |  | Increasing then decreasing trend | |  | Decreasing then increasing trend | |
| --- | --- | --- | --- | --- | --- | --- | --- | --- | --- | --- | --- |
| eGFR | |  | Increasing trend | | --- | --- | |  | Decreasing trend | |  | No significant change | |  | Increasing then decreasing trend | |  | Decreasing then increasing trend | |

3. Which of the following conditions require adjustment of treatment now?

|  | Diabetes |
| --- | --- |
|  | Cholesterol |

4. Which of the following test(s) is/are due for repeat testing now?

|  | HbA1c |
| --- | --- |
|  | LDL Cholesterol |

5. If they are not due now, when would you repeat them?

| HbA1c (in months) |  |
| --- | --- |
| LDL Cholesterol (in months) |  |

1. How confident are you in interpreting diabetes and lipid-related laboratory results now, using the given interface?

|  | 0 | 1 | 2 | 3 | 4 | 5 | 6 | 7 | 8 | 9 | 10 |  |
| --- | --- | --- | --- | --- | --- | --- | --- | --- | --- | --- | --- | --- |
| Not Confident |  |  |  |  |  |  |  |  |  |  |  | Very Confident |

Thank you for participating in our survey.
